# Synaptic Homeostasis and Allostasis in the Dentate Gyrus Caused by Inflammatory and Neuropathic Pain Conditions

**DOI:** 10.3389/fnsyn.2018.00001

**Published:** 2018-01-31

**Authors:** Rui-Rui Wang, Yan Wang, Su-Min Guan, Zhen Li, Saurabh Kokane, Fa-Le Cao, Wei Sun, Chun-Li Li, Ting He, Yan Yang, Qing Lin, Jun Chen

**Affiliations:** ^1^Institute for Biomedical Sciences of Pain, Tangdu Hospital, The Fourth Military Medical University, Xi’an, China; ^2^Key Laboratory of Brain Stress and Behavior, People’s Liberation Army, Xi’an, China; ^3^School of Stomatology, The Fourth Military Medical University, Xi’an, China; ^4^Department of Psychology, University of Texas at Arlington, Arlington, TX, United States; ^5^Department of Neurology, The 88th Hospital of People’s Liberation Army, Tai’an, China; ^6^Beijing Institute for Brain Disorders, Beijing, China

**Keywords:** synaptic homeostasis and allostasis, inflammatory pain, neuropathic pain, dentate gyrus, excitatory synaptic transmission, inhibitory synaptic modulation

## Abstract

It has been generally accepted that pain can cause imbalance between excitation and inhibition (homeostasis) at the synaptic level. However, it remains poorly understood how this imbalance (allostasis) develops in the CNS under different pain conditions. Here, we analyzed the changes in both excitatory and inhibitory synaptic transmission and modulation of the dentate gyrus (DG) under two pain conditions with different etiology and duration. First, it was revealed that the functions of the input-output (I/O) curves for evoked excitatory postsynaptic currents (eEPSCs) following the perforant path (PP) stimulation were gained under both acute inflammatory and chronic neuropathic pain conditions relative to the controls. However, the functions of I/O curves for the PP-evoked inhibitory postsynaptic currents (eIPSCs) differed between the two conditions, namely it was greatly gained under inflammatory condition, but was reduced under neuropathic condition in reverse. Second, both the frequency and amplitude of miniature IPSCs (mIPSCs) were increased under inflammatory condition, however a decrease in frequency of mIPSCs was observed under neuropathic condition. Finally, the spike discharge of the DG granule cells in response to current injection was significantly increased by neuropathic pain condition, however, no different change was found between inflammatory pain condition and the control. These results provide another line of evidence showing homeostatic and allostatic modulation of excitatory synaptic transmission by inhibitory controls under different pathological pain conditions, hence implicating use of different therapeutic approaches to maintain the homeostasis between excitation and inhibition while treating different conditions of pathological pain.

## Introduction

Recently, it has been suggested that pain should be redefined as “a distressing experience associated with actual or potential tissue damage with sensory, emotional, cognitive and social components” (Williams and Craig, 2016). Pain can be acute or chronic in duration. Acute pain is defined as the event of recent onset with probable limited duration that usually has an identifiable temporal and causal relationship to injury or disease (Merskey and Bogduk, 1994). However, unlike acute pain, chronic pain has unlimited duration (more than 3 months) that rarely has an identifiable temporal and causal relationship to injury or disease in the body (Merskey and Bogduk, 1994). Chronic conditions of clinical pain have also been newly classified into seven subcategories for the International Classification of Diseases version 11 (ICD-11) of the World Health Organization: (1) chronic primary pain; (2) chronic cancer pain; (3) chronic posttraumatic and postsurgical pain; (4) chronic neuropathic pain; (5) chronic headache and orofacial pain; (6) chronic visceral pain; and (7) chronic musculoskeletal pain based on etiology, region, comorbidities, pathophysiological processes, diagnosis and therapies (Treede et al., 2015). These actions of redefining pain and reclassifying chronic conditions of pain strongly implicate the importance of in-depth studying the mechanisms of different pain conditions.

It has been generally accepted that pain can cause imbalance between excitation and inhibition (homeostasis) at the synaptic level. However, it remains poorly understood how this imbalance (allostasis) develops in the CNS under different pain conditions. In our and others’ previous reports, the limbic system including the medial prefrontal cortex, the hippocampal formation (HF) and amygdala has been demonstrated to be highly involved in emotional, cognitive and social modulation of pain (Ren et al., 2008; Neugebauer et al., 2009; Lyu et al., 2013; Li et al., 2014; Neugebauer, 2015; Geng et al., 2016; Lu et al., 2016; for reviews see Liu and Chen, 2009, 2014). Synaptic plasticity at both excitatory and inhibitory synapses serves as an important universal mechanism underlying pain-associated emotional and cognitive deficits (Zhao et al., 2009; Gong et al., 2010; Liu et al., 2011, 2012; Lu et al., 2014; for reviews see Liu and Chen, 2009, 2014). Furthermore, the imbalance between excitation and inhibition is one of the major factors causing changes in net synaptic output of the neural circuits, which contributes to the pathophysiology of several diseases, including pain, epilepsy, depression and schizophrenia (de Lanerolle et al., 2003; Yan et al., 2009; Gong et al., 2010; Croarkin et al., 2011; Sanderson et al., 2012; Amakhin et al., 2016; for review see Liu and Chen, 2014). From the point of this view, contributions of the excitatory and inhibitory events to the modulation of pain have been considerably studied in the past decade. It has been suggested that various pain stimuli can induce the enhancement of the excitatory synaptic transmission in several brain regions, including the HF (Zhao et al., 2009; Liu et al., 2011, 2012; Lyu et al., 2013), amygdala (Ren and Neugebauer, 2010; Li et al., 2011), primary somatosensory cortex (Chang et al., 2008; Wang et al., 2010; Eto et al., 2012), anterior cingulate cortex (Zhao et al., 2006; Gong et al., 2010; Lu et al., 2014) and even primary motor cortex (Wang et al., 2011).

In addition to the enhancement of the excitatory synaptic transmission, changes in inhibitory synaptic modulation have also been demonstrated to be involved in both inflammatory and neuropathic pain conditions. At the spinal level, the number of GABA-immunoreactive cells has been shown to be increased in the ipsilateral dorsal horn under inflammatory pain condition that is dependent upon the on-going gain in primary nociceptive afferent (Castro-Lopes et al., 1994). However, contrarily a loss of inhibitory modulation in the spinal dorsal horn has been revealed under neuropathic pain condition (Dickenson et al., 1997). Electrophysiological recordings at the synaptic level also demonstrated dramatic decrease in GABA receptor-mediated tonic currents and pre-synaptic GABA release in the substantia gelatinosa after sciatic nerve chronic constriction injury (CCI) in mice (Iura et al., 2016). The results of those previous studies suggest that synaptic inhibition is enhanced in the inflammatory pain state but reduced in the neuropathic pain state in the CNS. However, how excitatory and inhibitory synaptic transmission and modulation would change at higher level of the CNS under different pain conditions remains largely unknown.

The HF, an integral component of the limbic system (MacLean, 1949; Papez, 1995) has been proposed to actively participate in the modulation of nociceptive stimuli (McKenna and Melzack, 1992; Zhao et al., 2009; Liu et al., 2011, 2012; Lyu et al., 2013). The dentate gyrus (DG) of the HF receives synaptic input to granule cells from the entorhinal cortex (EC) through the perforant path (PP) and sends synaptically-integrated information directly to the CA3 pyramidal cells, finally forming a typical triple EC-DG-CA3-CA1 synaptic circuitry (Liu and Chen, 2009). In our previous studies, we have demonstrated that the EC-DG synaptic connections can be significantly enhanced in both space and time by nociceptive information elicited by acute peripheral inflammatory pain produced by subcutaneous (s.c.) injection of bee venom (BV; Zhao et al., 2009; Liu et al., 2011, 2012; Lyu et al., 2013; for reviews see Liu and Chen, 2009, 2014). Specifically, multi-electrode array recordings with 64 channels (8 × 8) revealed distinct increase in the number of field potentials (spatial enlargement of network response) as well as temporal enhancement of long-term potentiation (LTP) under the BV-induced inflammatory condition (Zhao et al., 2009; Liu et al., 2011, 2012; Lyu et al., 2013). Nonetheless, it is interesting to note that the synaptic gain across the EC-DG synapses under acute inflammatory pain condition are regulated by different counteracting factors (Liu et al., 2011, 2012; Lyu et al., 2013). Namely, metabotropic glutamate receptor (mGluR) subtype 5 and mitogen-activated protein kinase (MAPK) members including extracellular signal-regulated kinase (ERK) and c-Jun N-terminal kinase (JNK) were shown to be involved in maintenance of pain-associated EC-DG synaptic enhancement (Zhao et al., 2009; Liu et al., 2011, 2012), whereas mGluR1 and p38 MAPK were playing counteracting roles against this synaptic enhancement through tonic inhibition (Zhao et al., 2009; Liu et al., 2011, 2012). Moreover, mammalian target of rapamycin (mTOR)-S6K signaling has also been demonstrated to mediate the EC-DG synaptic enhancement and pain-related anxiety-like behaviors (ALB, Lyu et al., 2013). However, how synaptic functional connections in the HF are affected by chronic neuropathic condition is yet known.

In the present study, we examined both the excitatory and inhibitory synaptic events in the DG to determine whether synaptic transmission and modulation is well maintained (synaptic homoestasis) or disrupted (synaptic allostasis) in response to acute inflammatory pain and chronic neuropathic pain. The BV model was used as acute inflammatory pain because the pain-related behaviors and hypersensitivity last within a limited period for utmost 72–96 h (Chen et al., 1999, 2006; Chen and Chen, 2000; Chen and Lariviere, 2010; Chen and Guan, 2017), while spared nerve injury (SNI) model was used as chronic neuropathic pain because pain hypersensitivity lasts for more than 6 months without recovery (Decosterd and Woolf, 2000). Using slice patch-clamp recording, we measured the changes in neuronal excitability, synaptic transmission, and synaptic plasticity on freshly prepared hippocampal slices under both BV- and SNI-induced pain conditions.

## Materials and Methods

### Animals

All experiments were carried out on male Sprague-Dawley rats (weighing from 80 g to 100 g) obtained from the Laboratory Animal Center of the Fourth Military Medical University (FMMU). The animals were kept under controlled conditions of temperature (25–26°C) and circadian cycle (12 h light/12 h dark) with *ad libitum* access to food and water. All experimental procedures were approved by the Institutional Animal Care and Use Committee of FMMU (No. 20150202) and were consistent with the ethical guidelines of the International Association for the Study of Pain for Pain Research in Conscious Animals (Zimmermann, 1983). Efforts were made to minimize the number of animals used and their sufferings.

### Animal Models of Different Pain Conditions

#### Inflammatory Pain Model

Lyophilized whole venom of *Apis mellifera* (Sigma, St. Louis, MO, USA) dissolved in 0.9% sterile saline was used. For the BV-inflamed group, a volume of 50 μl saline containing 0.2 mg BV was used during the whole experiment (Chen et al., 1999). Subcutaneous injection of BV was administered into the posterior plantar surface of the left hind paw of rats as reported previously (Chen et al., 1999). For the saline group, rats received an equal volume of sterile saline.

#### Neuropathic Pain Model

The SNI model was induced according to the protocol described previously (Decosterd and Woolf, 2000). Rats were anesthetized with sodium pentobarbital (50 mg/kg, i.p.). The three branches of the sciatic nerve were exposed above the knee. The tibial and common peroneal branches were tightly ligated and severed while leaving the sural nerve intact. For sham surgery, the three sciatic branches were also exposed but then left intact. Mechanical allodynia was tested on post-surgical day 3 and the rats were then used for electrophysiological recordings on post-surgical days 6–7. All the rats were left undisturbed in their home cage until brain slice preparation was conducted.

### Hippocampal Slice Preparation

The method used for obtaining brain slice of the HF was similar to that described elsewhere (Zhao et al., 2009). Briefly, rats were anesthetized with 25% urethane (1.2 g/kg, i.p.) at 2 h after BV injection or 6–7 days after SNI surgery and sacrificed by decapitation. Subsequently, the brain was rapidly removed and placed in ice-cold artificial cerebrospinal fluid (ACSF) pre-equilibrated with 95% O_2_ and 5% CO_2_ (in mM: NaCl 117, KCl 3.6, NaH_2_PO_4_·2H_2_O 1.2, MgCl_2_·6H_2_O 1.2, CaCl_2_·2H_2_O 2.5, NaHCO_3_ 25, glucose 11, adjusted to 285–295 mOsm, pH 7.3). After cooling for about 2 min, the tissue blocks containing the hippocampus of right hemisphere (i.e., contralateral to the BV injection side or the SNI surgery side) were immediately dissected and glued to the stage of a vibratome (DTK-1000, Dosaka EM. CO. LTD., Japan) that was filled with preoxygenated ice-cold ACSF. Two to three transverse slices, including the region of the HF, were cut at a thickness of 300 μm each. Each slice was transferred to a holding chamber with oxygenated ACSF (95% O_2_ and 5% CO_2_) and maintained at room temperature for at least 2 h for recovery before starting electrophysiological recordings (Zhao et al., 2009; Gong et al., 2010).

### Electrophysiological Recordings

**Figure 1:**
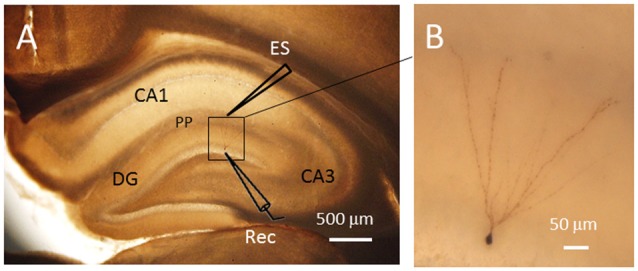
Single cell labeling with biocytin after electrophysiological recordings. The labeled neuron showed typical morphological characteristics of granule cells in the dentate gyrus (DG). **(A)** The locations of the electrodes for electrical stimulation (ES) and patch clamp recording (Rec). **(B)** Amplified image of the labled neuron within the DG. The soma of the granule cell recorded has a diameter of approximately 7 μm. PP, perforant path fibers. Scale bars = 500 μm for **(A)** and 50 μm for **(B)**.

For patch clamp recording, a single brain slice was held down in the recording chamber with an anchor (SHD-22L, Harvard, Cambridge, MA, USA) and was kept immersed in circulating oxygenated ACSF at a flow rate of 2.2–2.6 ml/min using a fast perfusion system (Peri-star, WPI, Worcester, MA, USA). Postsynaptic currents were recorded from granule cell of the DG. Under voltage-clamp mode, the membrane potential was clamped at −70 mV in order to maintain physiological conditions. The neurons recorded were visualized with an infrared video microscope (BX51WI, Olympus, Japan). Whole-cell recordings were made from granule cells of the DG (Figure 1). The patch electrodes (2–4 MΩ) were prepared using borosilicate tubing (1.5 mm outside diameter, 0.86 mm inside diameter, Nanjing, China) on a vertical microelectrode puller (PC-10, Narishiga, Japan). A bipolar tungsten-stimulating electrode was positioned in the PP fiber bundle, and a single pulse was delivered at 0.05 Hz (10 μs, 0.3–0.7 mA) with a 0.1 mA stepwise increase. The electrodes were filled with an internal solution containing (in mM): Cs_2_SO_4_, 110; CaCl_2_, 0.5; MgCl_2_, 2; EGTA, 5; HEPES, 5; tetraethylammonium (TEA), 5; with pH adjusted to 7.2–7.4 by CsOH. The chloride equilibrium potential was −83 mV that is calculated by Nernst Equation. The osmolarity is between 290 mOsm and 320 mOsm. Biocytin was added to the intracellular solution for neuronal labeling. A typical granule cell labeled with biocytin was shown in Figure 1. To record glutamate receptor mediated excitatory postsynaptic events such as evoked excitatory postsynaptic currents (eEPSCs) and miniature EPSCs (mEPSCs), whole-cell recordings were carried out on DG granule cells under voltage-clamp at −70 mV in the presence of 10 μM bicuculline, an GABA_A_ receptor antagonist added to ACSF so as to block GABA_A_-receptor-mediated inhibitory postsynaptic currents (IPSCs). To record GABA receptor-mediated IPSCs such as evoked IPSCs (eIPSCs) and miniature IPSCs (mIPSCs), whole-cell recordings were carried out under voltage-clamp at 0 mV in the presence of ionic glutamate receptor antagonists including CNQX against α-amino-3-hydroxy-5-methyl-4-isoxazole propionic acid (AMPA) receptor and D-(-)-2-amino-5-phosphonopentanoic acid (D-APV) against N-methyl-D-aspartic acid (NMDA) receptor. Recordings of mEPSCs and mIPSCs were carried out in the presence of 1 μM tetrodotoxin (TTX), a non-selective sodium channel blocker, so as to block spike activity-dependent EPSCs and IPSCs. Series resistance was in the range of 20–30 MΩ. Data were discarded if the resistance changed >30% during recording or the resting membrane potential (RMP) of the neurons was depolarized at voltages higher than −50 mV. The frequency and amplitude of mEPSCs and mIPSCs were measured using MiniAnalysis (Synaptosoft, Decatur, GA, USA^1^). Current signals were recorded with an EPC 10 series patch clamp amplifier (Molecular Devices, German). Data were digitized and stored on disks using PULSE acquisition and analysis software (Molecular Devices, Germany). Spike discharge was recorded at the current clamp mode and postsynaptic currents were recorded at the voltage-clamp mode. Detailed methodology has been described in our previous reports (Gong et al., 2010; Wang et al., 2014).

### Drug Application

All drugs were purchased from Sigma Aldrich (St. Louis, MO, USA). Bicuculline (10 μM), CNQX (10 μM), D-APV (50 μM) and TTX (1 μM) were dissolved in the ACSF to their final concentration on the day of the experiment and applied to the brain slices with perfusion when tested. Selectivity and target concentrations were chosen based on preliminary experiments and previous reports (Gong et al., 2010; Wang et al., 2014).

### Data Acquisition and Statistical Analysis

The frequency and amplitude of the postsynaptic events were calculated using pCLAMP 9.0 and Mini Analysis v6.09. Data were analyzed offline by MiniAnalysis Software. In brief, the detection threshold was set based on the root mean square (RMS) noise level. Miniature postsynaptic currents were first detected automatically by the software using an amplitude threshold of 10.6 ± 0.53 pA and an area threshold of 13.2 ± 0.8 fC. All events were then visually reexamined. Any noise that spuriously met the trigger specifications was rejected. Independent *t*-test was adopted as the statistical method for the frequency and amplitude of the postsynaptic events. The cumulative probability distribution of inter-event interval (ms) and amplitude (pA) for mEPSCs and mIPSCs were assessed by the Kolmogorov-Smirnov test. A level of *p* values less than 0.05 was accepted as significance. All data were expressed as means ± SEM.

## Results

### Excitatory Synaptic Transmission in the DG Was Enhanced by Both Inflammatory and Neuropathic Pain Conditions

**Figure 2:**
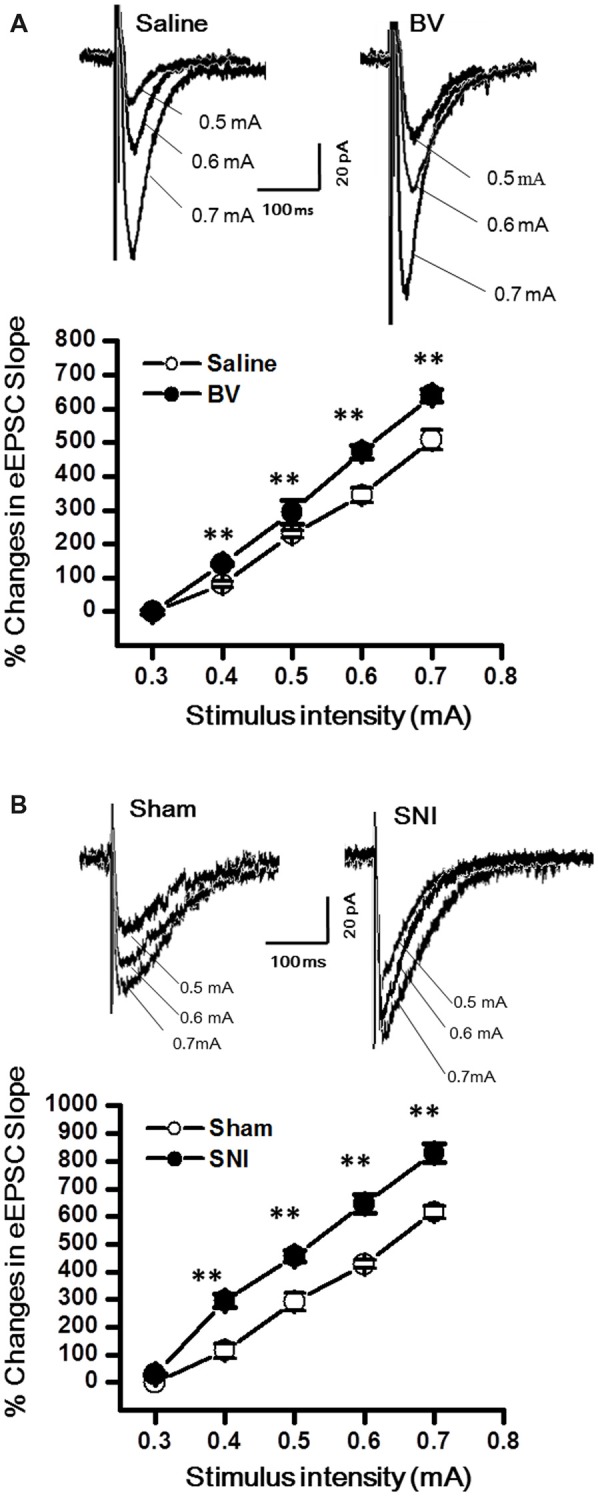
Evoked excitatory postsynaptic currents (eEPSCs) under the bee venom (BV)-induced inflammatory pain condition **(A)** and the spared nerve injury (SNI)-induced neuropathic pain condition **(B)**. **(A)** Input-output (I/O) function of monosynaptic eEPSCs in slices from BV-inflamed and Saline groups. **(B)** I/O function of monosynaptic eEPSCs in slices from SNI and Sham groups. The representative traces of eEPSCs for both **(A,B)** were obtained by stimulus intensities of 0.5, 0.6 and 0.7 mA, respectively. **p < 0.01; *V*_H_ = −70 mV.

The DG granule neurons were initially identified using IR-DIC by their elliptic shaped cell bodies with a width of approximately 10 μm and a height of 18 μm and confirmed *post hoc* using single cell labeling with biocytin (Claiborne et al., 1990; Figure 1). To examine whether there was a change in excitatory synaptic transmission for EC-DG synapses under different pain conditions (BV vs. SNI), eEPSCs were recorded from the DG granule neurons following electrical stimuli at the PP (Figure 2). The input-output (I-O) relationships were obtained by measuring the slopes of eEPSCs in response to ascending stepwise intensities of electrical stimulus (0.3–0.7 mA, 0.1 mA stepwise). Under both BV-induced inflammatory and SNI-induced neuropathic pain conditions, the I/O curves shifted leftward relative to the controls (Figure 2A for BV vs. Saline and Figure 2B for SNI vs. Sham, **p < 0.01), implicating an enhancement of synaptic transmission for the EC-DG synapses (Figure 2). The slope of I/O curve for BV group (253.9 ± 8.5 pA/V, *n* = 6 cells/6 rats) was much steeper than the Saline control group (170.9 ± 8.8 pA/V, *n* = 6 cells/6 rats; *p* < 0.01, lower panel of Figure 2A). So was steeper for the slope of I/O curve in the SNI group (177.2 ± 7.5 pA/V, *n* = 6 cells/6 rats) than in the Sham control (129.4 ± 4.4 pA/V, *n* = 10 cells/7 rats; *p* < 0.01, lower panel of Figure 2B).

**Figure 3:**
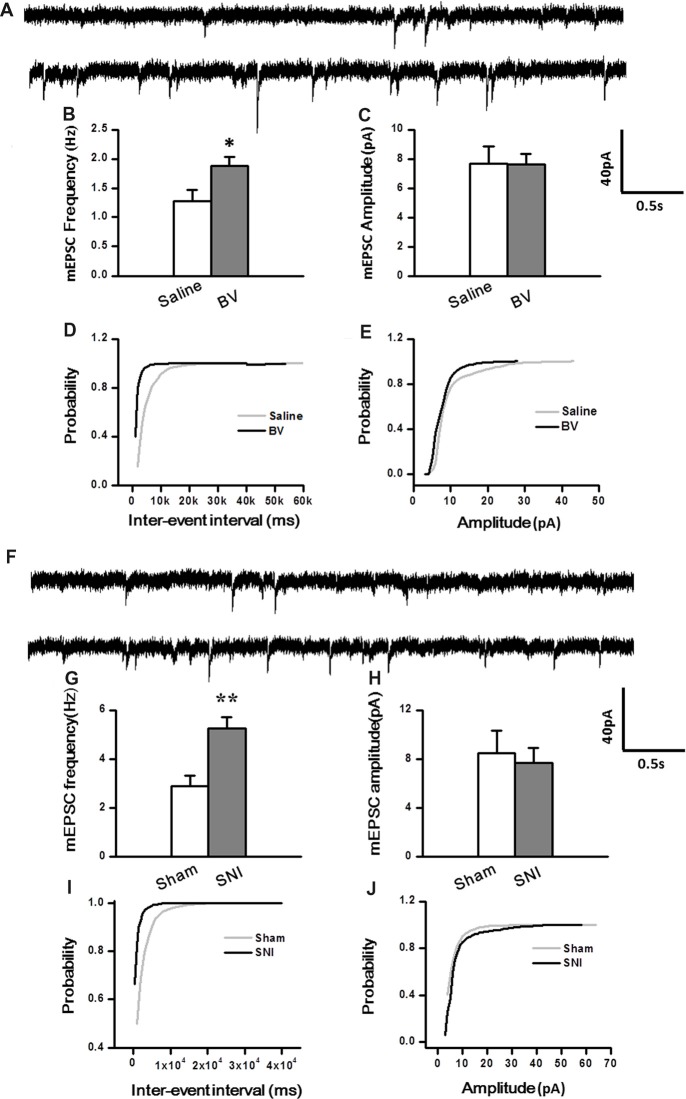
Miniature excitatory postsynaptic currents (mEPSCs) under the BV-induced inflammatory pain condition **(A–E)** and the SNI-induced neuropathic pain condition **(F–J)**. **(A)** Representative traces of mEPSCs from DG neurons of Saline-control (*top*) and BV-inflamed rats (*lower*). **(B)** Frequency and **(C)** amplitude of mEPSCs. The cumulative distribution probability curves for inter-event intervals **(D)** and amplitude **(E)** of mEPSCs. **(F)** Representative traces of mEPSCs of Sham (*top*) and SNI-treated rats (*lower*). **(G)** Frequency and **(H)** amplitude of mEPSCs. The cumulative distribution probability curves for inter-event intervals **(I)** and amplitude **(J)** of mEPSCs. *p < 0.05; **p < 0.01; *V*_H_ = −70 mV.

To determine whether intrinsic properties of excitatory presynaptic and postsynaptic components are also changed in the DG under different pain conditions, mEPSCs were also recorded. The frequency of mEPSCs increased significantly in the BV group (BV vs. saline group: 1.9 ± 0.2 Hz, *n* = 7 cells/6 rats vs. 1.3 ± 0.2 Hz, *n* = 7 cells/6 rats, *p* < 0.05, Figures 3A,B). However, the amplitude of mEPSCs remained unchanged in BV-inflamed rats (BV vs. saline groups: 7.6 ± 0.7 pA vs. 7.7 ± 1.2 pA, *p* = 0.97, Figures 3A,C). The cumulative probability distribution curve of the mEPSCs inter-event interval for the BV group shifted leftward to that of the Saline group (Kolmogorov-Smirnov test, *p* < 0.05, Figure 3D), while that of the mEPSCs amplitude remained relatively unchanged (Kolmogorov-Smirnov test, *p* = 0.15, Figure 3E). Similar results were found in the SNI group, the frequency of mEPSCs also increased significantly (SNI vs. Sham group: 5.24 ± 0.47 Hz, *n* = 7 cells/6 rats vs. 2.9 ± 0.44 Hz, *n* = 7 cells/6 rats, *p* < 0.01, Figures 3F,G). There was no significant difference in the amplitude between SNI-treated rats and sham-control rats (SNI vs. Sham group: 7.71 ± 1.21 pA vs. 8.51 ± 1.81 pA, *p* = 0.72, Figures 3F,H). The cumulative probability distribution curve of the mEPSCs inter-event interval in the SNI group also shifted leftward to that of the sham group (Kolmogorov Smirnov test, *p* < 0.05, Figure 3I), while that of the mEPSCs amplitude remained unchanged either (Kolmogorov Smirnov test, *p* = 0.59, Figure 3J).

### Inhibitory Synaptic Transmission in the DG Was Enhanced by Inflammatory Pain but Decreased by Neuropathic Pain Condition

**Figure 4:**
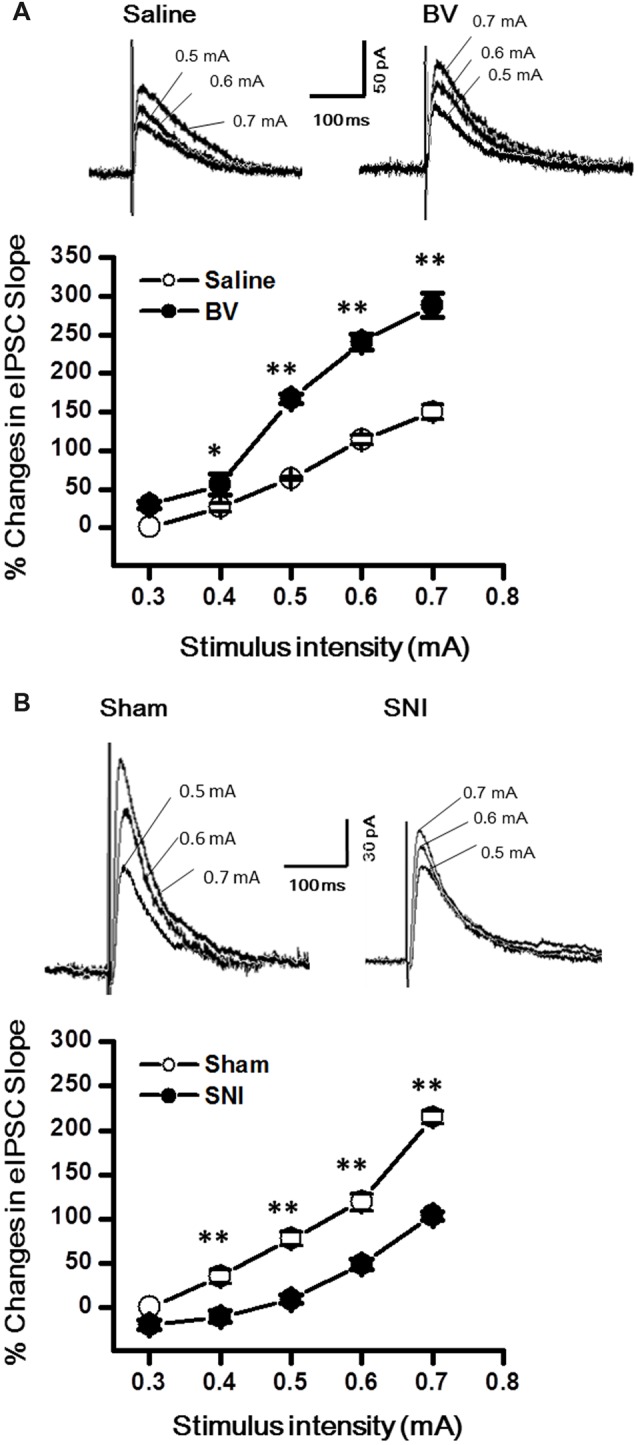
Evoked inhibitory postsynaptic currents (eIPSCs) under the BV-induced inflammatory pain condition **(A)** and the SNI-induced neuropathic pain condition **(B)**. **(A)** I/O function of eIPSCs in BV and Saline groups. **(B)** I/O function of eIPSCs in SNI and Sham groups. The representative traces of eIPSCs for both **(A,B)** were obtained by stimulus intensities of 0.5, 0.6 and 0.7 mA, respectively. *p < 0.05, **p < 0.01; *V*_H_ = 0 mV.

Synaptic modulation in the hippocampus plays a crucial role in balancing and synchronizing the activities of excitatory cells. To examine whether there was a change in inhibitory modulation for EC-DG synapses under different pain conditions (BV vs. SNI), eIPSCs were recorded from the DG granule neurons following electrical stimuli at the PP (Figure 4). The I/O curves were obtained by measuring the slopes of eIPSCs in response to ascending stepwise intensities of electrical stimulus (0.3–0.7 mA, 0.1 mA stepwise). Unlike the results for eEPSCs, the I/O curve of eIPSCs for the BV group shifted leftward to the Saline controls (Figure 4A for BV vs. Saline, *p < 0.05; **p < 0.01), while conversely that of eIPSCs for the SNI group shifted rightward to the Sham group (Figure 4B for SNI vs. Sham, **p < 0.01), implicating that different pain conditions have different influences on the inhibitory synaptic modulation of the EC-DG synapses (Figure 4). The slope of I/O curve for the BV group (253.3 ± 11.0 pA/V, *n* = 8 cells/5 rats) was much steeper than the Saline control group (127 ± 16.1 pA/V, *n* = 8 cells/5 rats; *p* < 0.01, lower panel of Figure 4A), whereas, that for the SNI group (145.6 ± 8.7 pA/V, *n* = 11 cells/7 rats) was less steep than the Sham group (221 ± 8.6 pA/V, *n* = 10 cells/6 rats; *p* < 0.01, lower panel of Figure 4B).

**Figure 5:**
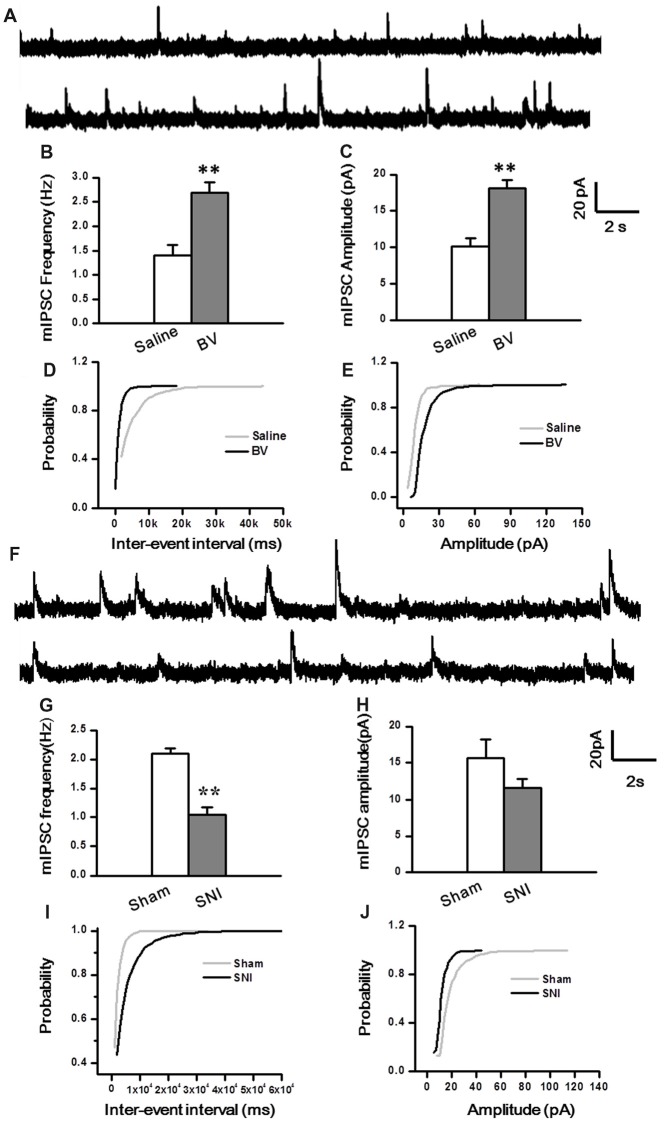
Miniature inhibitory postsynaptic currents (mIPSCs) under the BV-induced inflammatory pain condition **(A–E)** and the SNI-induced neuropathic pain condition **(F–J)**. **(A)** Representative traces of mIPSCs in the DG neurons of Saline (*top*) and BV (*lower*). **(B)** Frequency and **(C)** amplitude of mIPSCs. The cumulative distribution probability curve for inter-event intervals **(D)** and amplitude **(E)** of mIPSCs. **(F)** Representative traces of mIPSCs in Sham (*top*) and SNI (*lower*) groups. **(G)** Frequency and **(H)** amplitude of mIPSCs. The cumulative distribution probability curves for inter-event intervals **(I)** and amplitude **(J)**. **p < 0.01; *V*_H_ = 0 mV.

To determine whether intrinsic properties of inhibitory presynaptic and postsynaptic components are also differently changed in the DG by different pain conditions, mIPSCs were also recorded (Figure 5). As shown in Figures 5A–E, both frequency and amplitude of mIPSCs were significantly increased in the BV group as compared with the saline control (Frequency for BV vs. Saline: 2.7 ± 0.2 Hz, *n* = 7 cells/6 rats vs. 1.4 ± 0.2 Hz, *n* = 6 cells/6 rats; Amplitude for BV vs. Saline: 18.1 ± 1.1 pA vs. 10.1 ± 1.1 pA, see Figures 5B,C). The mean area under the curve (AUC) of mIPSCs for the saline group was 210 ± 6.3 nC (2573 events in total, *n* = 6 cells/6 rats), while that for the BV group was 274.2 ± 13.6 nC (3158 events in total, *n* = 7 cells/6 rats), showing a significant difference between the two groups (*p* < 0.01). Consistent with the above data, the cumulative probability distribution curve of the mIPSC inter-event interval for the BV group shifted leftward to that of the Saline group (Kolmogorov Smirnov test, *p* < 0.05, Figure 5D), while that of the mIPSCs amplitude for the BV group shifted rightward to the Saline control (Kolmogorov Smirnov test, *p* < 0.05, Figure 5E). In sharp contrast (Figures 5F–J), the frequency of mIPSCs in the SNI group was significantly decreased relative to the sham group (SNI vs. Sham: 1.05 ± 0.13 Hz, *n* = 8 cells/6 rats vs. 2.1 ± 0.09 Hz, *n* = 9 cells/7 rats, *p* < 0.01, Figure 5G). However, the amplitude of mIPSCs did not differ significantly between the two groups (SNI vs. Sham: 11.61 ± 1.22 pA vs. 15.7 ± 2.5 pA, *p* = 0.17, Figure 5H). The mean AUC of mIPSCs for the sham group was 221.1 ± 23 nC (1877 events in total, *n* = 9 cells/7 rats), while that for the SNI group was 227.8 ± 24 nC (1461 events in total, *n* = 8 cells/6 rats), showing no statistical significance between the two groups (*p* = 0.851). The accumulative probability distribution curve of the mIPSCs inter-event interval in the SNI group shifted rightward to that of the Sham group (Kolmogorov Smirnov test, *p* < 0.05, Figure 5I), while the probability distribution of mIPSCs amplitude was not statistically different between the two groups (Kolmogorov Smirnov test, *p* = 0.42, Figure 5J).

### Excitability of DG Granule Cells Was Not Altered by Inflammatory Pain, but Was Increased by Neuropathic Pain Condition

**Figure 6:**
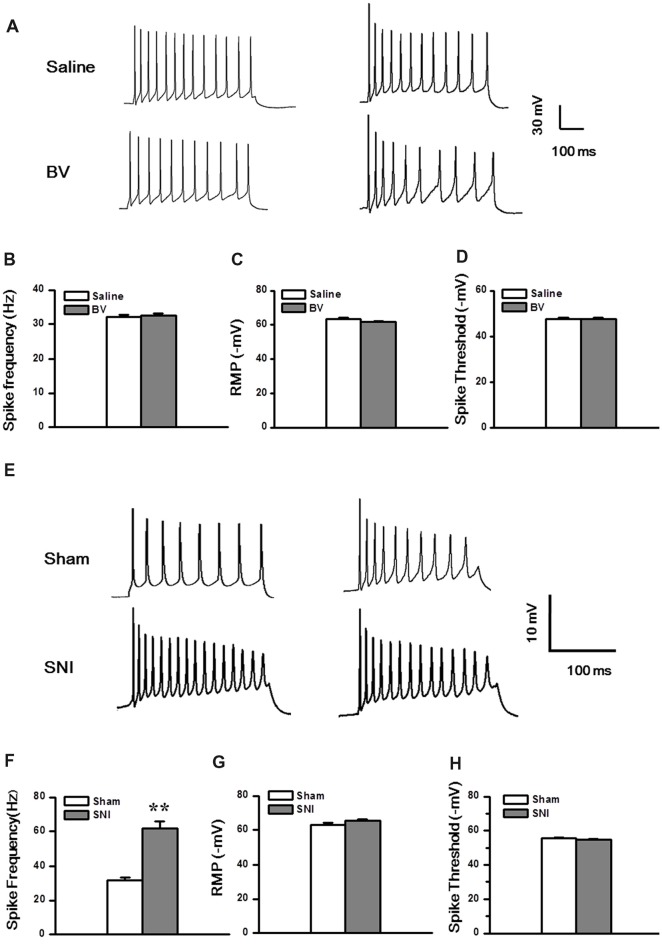
Membrane property and excitability in the DG neurons under the BV-induced inflammatory pain condition **(A–D)** and the SNI-induced neuropathic pain condition **(E–H)**. **(A)** Representative traces of spike discharges of two neurons from both Saline and BV groups in response to a stimulus intensity of 100 pA. Bar graphs show spiking frequency **(B)**, resting membrane potentials (RMP) **(C)**, and spike threshold **(D)** of the neurons. **(E)** Representative traces of spike discharges of another two neurons from both Sham and SNI groups in response to a stimulus intensity of 100 pA. Bar graphs show spiking frequency **(F)**, RMP **(G)**, and spike threshold **(H)** of the neurons. **p < 0.01, SNI vs. Sham.

Next, we explored whether the excitability of the DG neurons was changed under the two different pain conditions (Figures 6A–H). Under the current clamp model (Figure 6A), the spike discharge frequency of the DG neurons did not differ between the Saline and the BV groups in response to 100 pA current stimulation (32.1 ± 0.8 Hz, *n* = 13 cells/7 rats vs. 32.6 ± 0.6 Hz, *n* = 9 cells/6 rats, *p* = 0.6, Figure 6B). Neither the RMPs (Saline vs. BV: −63.6 ± 0.8 mV vs. −61.8 ± 0.5 mV, *p* = 0.09, Figure 6C) nor the spike thresholds (Saline vs. BV: −47.6 ± 0.8 mV vs. −47.4 ± 0.8 mV; *p* = 0.9, Figure 6D) was significantly different between the BV and control groups for inflammatory pain condition.

Under neuropathic pain condition, in contrast, a significant increase in spike firing rate was observed in the DG neurons of the SNI group as compared with the Sham control (Sham vs. SNI: 32.1 ± 0.8 Hz, *n* = 8 cells/6 rats vs. 61.8 ± 4.1 Hz, *n* = 9 cells/7 rats, *p* < 0.01; Figures 6E,F). However, neither the RMP (Sham vs. SNI, −63.2 ± 0.9 mV vs.–65.3 ± 0.8 mV, *p* = 0.09; Figure 6G) nor the spike threshold was significantly changed under neuropathic pain condition (Sham vs. SNI, −55.7 ± 0.3 mV vs. −54.9 ± 0.5 mV, *p* = 0.23; Figure 6H).

## Discussion

**Figure 7:**
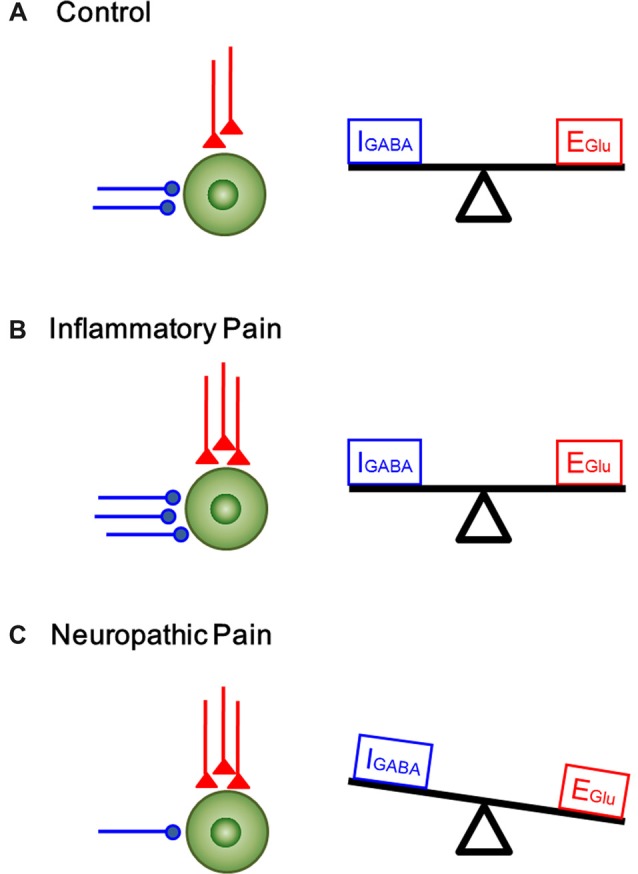
A proposed homeostatic and allostatic model of excitatory/inhibitory synaptic transmission and modulation under inflammatory pain and neuropathic pain conditions. The inhibitory (I_GABA_) and excitatory (E_Glu_) inputs are denoted in blue and red respectively. **(A)** A balanced state of excitatory and inhibitory synaptic inputs under physiological condition. **(B)** The increase in excitatory input in the DG was accompanied by corresponding rise in inhibitory control under inflammatory pain condition (synaptic homoestasis). **(C)** Peripheral nerve injury resulted in decrease in inhibitory synaptic control and increase in excitatory synaptic transmission, leading to imbalance between excitatory transmission and inhibitory control (synaptic allostasis).

In the current study, we analyzed the functional changes in both excitatory and inhibitory synaptic transmission and modulation in the DG under two pain conditions with different etiology and duration. Under acute peripheral inflammatory pain condition, the function of inhibitory synaptic modulation in the DG was gained (seen as leftward shift of eIPSCs I/O curve) in response to increased excitatory synaptic transmission (also seen as leftward shift of eEPSCs I/O curve). The increased excitatory synaptic transmission in the DG has been demonstrated to be activity-dependent because peripherally local administration of bupivacaine or lidocaine with BV solution could block pain, pain-related ALB as well as the enhanced synaptic transmission in the DG (Zhao et al., 2009; Lyu et al., 2013). Recordings of mEPSCs in the DG following s.c. BV treatment only revealed increase in frequency, but not amplitude, suggesting that only presynaptic glutamate release is enhanced but with postsynaptic mechanisms being relatively unchanged. However, both frequency and amplitude of mIPSCs in the DG were increased following s.c. BV injection, implicating involvement of both presynaptic and postsynaptic functions of inhibitory GABAergic modulation counteracting against the gain of excitatory synaptic input, resulting in synaptic homeostasis under acute peripheral inflammatory pain condition (see Figure 7B). Under chronic peripheral neuropathic pain condition, however, the function of inhibitory synaptic modulation in the DG was reduced (seen as rightward shift of eIPSCs I/O curve) in response to increased excitatory synaptic transmission (seen as leftward shift of eEPSCs I/O curve). Under the same condition, the frequency of mIPSCs in the DG was reduced as well, while that of mEPSCs was increased in reverse. The amplitude of either mIPSCs or mEPSCs remained relatively unchanged. Together, these results suggest that GABAergic modulation in the SNI condition (long-term modification) is not anymore able to compensate synaptic excitation increase as in the BV condition (short term modification; Figure 7C).

The determinant factors that contribute to the different consequences in GABAergic synaptic modulation in the DG caused by the two pain conditions are less known and require to be further studied. The BV model differs from the SNI model in both etiology (tissue vs. nerve injury) and duration (hours vs. months). Because allostatic GABAergic modulation (seen as reduced mIPSCs with increased mEPSCs) has also been seen in the amygdala 3–7 days after s.c. injection of complete Freund’s adjuvant (CFA), another inflammatory model of pain (Chen et al., 2013), etiological factors are not likely to be more important than the duration of pain. The BV test is a well-established pain model consisting of both spontaneously pain-related paw flinches and primary mechanical/thermal hypersensitivity that naturally recovered within 72–96 h (Chen et al., 1999, 2006; Chen and Chen, 2000; Chen and Lariviere, 2010; Chen and Guan, 2017). However, the SNI preparation is a chronic neuropathic pain model with mechanical pain hypersensitivity lasting for more than 6 months without recovery in nature (Decosterd and Woolf, 2000). Based upon the results of the current study, it would be of special intriguing to know when is the turning point for transition from homeostatic modulation to allostatic modulation at synaptic level.

It has been known that the HF, typically established as the critical brain region processing learning and memory, is involved in the multi-dimensional processing of pain (Zhao et al., 2009; Liu et al., 2011, 2012; Lyu et al., 2013; for reviews see Liu and Chen, 2009, 2014). The HF is composed of two regions, the DG and *Cornu Ammonis* (CA). Granule cells in the DG sub-region receive synapses from neurons of the EC through the PP. The DG not only receives nociceptive afferent, but also plays an important role in nociceptive modulation (Zhao et al., 2009; Liu et al., 2011, 2012; Xu et al., 2012; Lyu et al., 2013). Electrophysiological recordings have shown that BV-induced pain-related experience in the periphery is capable of causing both spatial and temporal plasticity of synaptic connections, transmissions and functions in the HF (Zhao et al., 2009). Previous studies in humans also confirmed these observations by showing that the HF, together with other cortical structures, is involved in processing of noxious stimulation (Delgado, 1955; Halgren et al., 1978). Moreover, local injection of anesthetics in the DG produced anti-nociceptive effects in the formalin test (Klamt and Prado, 1991; McKenna and Melzack, 1992). Taken together, the results of those previous studies suggest that the HF plays a critical role in pain processing and provides convergent evidence that long-term, intense and complicated changes occur in synaptic plasticity under the condition of peripheral persistent nociception.

As the major excitatory component in the neuronal circuits, glutamatergic transmission participates in a variety of CNS functions, including pain modulation (Zhao et al., 2006, 2009; Gong et al., 2010). Consistent with our current results, it has been demonstrated that the enhancement of excitatory synaptic transmission in the ACC by the CFA treatment is associated with enhanced pre-synaptic neurotransmitter release but not postsynaptic mechanisms (Zhao et al., 2006). Moreover, the AMPAR-mediated glutamatergic transmission was enhanced with increased GluR1 expression and activity (Bie et al., 2011). The enhanced excitatory synaptic transmission has also been observed in the amygdala in both arthritis and CFA pain models (Ren and Neugebauer, 2010; Chen et al., 2013). Similarly, the enhancement of excitatory synaptic transmission in the ACC has also been reported to be caused by peripheral nerve injury (Xu et al., 2008; Toyoda et al., 2009). Together, it is likely that both inflammatory and neuropathic conditions cause enhancement of excitatory synaptic transmission in the CNS.

GABA has been well established as the major inhibitory neurotransmitter in the CNS. GABAergic modulation of nociceptive transmission has been shown to be involved in regulating pain information at the spinal level (Dickenson et al., 1997). Carrageenan-induced unilateral inflammation has also been shown to increase the number of GABA-immunoreactive cells in the ipsilateral dorsal horn of the spinal cord (Castro-Lopes et al., 1994). Similarly, in the S1, one of the principal pain-related brain regions (Guo et al., 2007; Liu et al., 2007; Chang et al., 2008; for review see Liu and Chen, 2014), the increased activities of both excitatory neurons and inhibitory neurons were observed under inflammatory pain condition (Eto et al., 2012). Synaptic inhibition in the hippocampus also plays a crucial role in balancing and synchronizing the activities of excitatory cells (Gutiérrez and Heinemann, 2006). As a new line of supporting evidence here, we demonstrated that both the frequency and amplitude of mIPSCs were increased in response to increased excitatory synaptic input induced by the BV injection. The parallel enhancement of excitatory and inhibitory synaptic transmission results in less change in membrane potential and excitability of the DG cells in the inflammatory pain state. Whereas, our findings that the frequency of mIPSCs was reduced but the amplitude remained relatively unchanged in the DG in the SNI model is indicative of a reduction in presynaptic GABA release under chronic neuropathic pain condition. This neuropathic pain-associated weakened inhibitory synaptic modulation has also been evidenced by another previous report showing reduction in the presynaptic GABA release in a mouse model of CCI (Iura et al., 2016).

GABAergic inhibitory system plays an important role in controlling the excitability and responsiveness of neurons in the CNS. In the hippocampus, the DG cells are mainly excitatory glutamatergic; however, the presence of inhibitory neurotransmitters in the mossy fiber terminals of granule cells raised a question whether the GABAergic feedback modulation of the DG cells via recurrent innervation exists so as to defend against stressful input under chronic pain conditions (Gutiérrez and Heinemann, 2006). In our present study, we found that inflammatory pain resulted in a parallel increase in the excitatory and inhibitory post-synaptic currents in the DG. The enhanced GABA appeared to play a role in controlling noxious transmission and so the extent of excitatory transmission is held in check. These findings suggest that the hippocampus can maintain synaptic homeostasis in response to BV-induced inflammatory pain. By contrast, we observed a counteracting change between excitatory synaptic transmission and inhibitory synaptic modulation in the DG under the neuropathic pain condition. After 6–7 days post-nerve injury, the inhibitory control failed to balance the excess of excitation, and the balance shifted toward excitatory events. Figure 7 shows a proposed model of homeostatic/allostatic regulation of excitatory/inhibitory synaptic transmission in the DG induced by different pain conditions. This homeostatic/allostatic states of synaptic modulation in the DG or other brain regions are likely to be associated with emotional comorbidity (ALB and depression-like behaviors) observed under inflammatory and/or neuropathic pain conditions.

Collectively, these findings indicate that SNI-induced neuropathic pain caused reduction in inhibitory control, while BV-induced inflammatory pain significantly increased the inhibitory synaptic modulation in the DG, suggesting an opposite regulation of GABAergic control between neuropathic pain and inflammatory pain states. These results provide evidence that different conditions of pathological pain show different properties in E/I balance at synaptic level, hence implicating that different therapeutic approach targeting at the synaptic level should be adopted toward different conditions of pathological pain.

## Author Contributions

JC conceived and designed the study. R-RW, YW, ZL, FL-C, C-LL, YY and TH performed the experiments. R-RW and WS analyzed the data. R-RW and JC drafted the manuscript. JC, QL, S-MG and SK revised the manuscript content. All authors read and approved the final manuscript.

## Conflict of Interest Statement

The authors declare that the research was conducted in the absence of any commercial or financial relationships that could be construed as a potential conflict of interest.

## Footnotes

^1^http://www.synaptosoft.com/MiniAnalysis/
